# Seed transmission of *Carlavirus vignae* (*Cowpea mild mottle virus*): a hidden driver of veinal necrosis and bud blight disease in soybean (*Glycine max*) in India

**DOI:** 10.3389/fmicb.2025.1654471

**Published:** 2025-09-16

**Authors:** Dhruva Nitin Bhagwatkar, Nagamani Sandra, Ankita Tripathi, Garima Dalal, Sharankumar Kesaratagi, Manisha Saini, Sandeep Kumar Lal, Sanjay Kumar Lal

**Affiliations:** ^1^Seed Pathology Laboratory, Division of Seed Science and Technology, ICAR-Indian Agricultural Research Institute, New Delhi, India; ^2^Division of Genetics, ICAR-Indian Agricultural Research Institute, New Delhi, India

**Keywords:** cowpea mild mottle virus, soybean, seed transmission, seed quality, veinal necrosis and bud blight disease

## Abstract

A comprehensive investigation was conducted to determine the seed transmission potential of cowpea mild mottle virus (CPMMV) associated with veinal necrosis and bud blight (VNB) disease in soybean (*Glycine max*) under Indian agroecological conditions during 2024 at ICAR-IARI, New Delhi. Seeds were collected from two CPMMV infected soybean genotypes, Asb-114 and AMS-2022-1 and mechanically sap inoculated cowpea genotypes Arka Samrudhi and Arka Suman. Serological testing of soybean and cowpea seeds using DAC-ELISA did not detect the virus with low absorbance values whereas RT-PCR with coat protein (CP)-specific primers confirmed the presence of CPMMV in whole seed, seed coat, cotyledons, and embryo with the amplification of 867 bp region. Grow out assays demonstrated the vertical transmission of CPMMV to the F₁ (75%) and F₂ (100%) progenies with the symptoms of necrotic spots and confirmed through RT-PCR and RT-qPCR. In parallel, cowpea genotypes were also confirmed for CPMMV infection in leaves, whole seed and seed parts by RT-PCR indicating the seed borne nature of the virus. Quantitative analysis using RT-qPCR revealed the highest viral titers in field infected soybean seeds (9.83 × 10^4^–5.35 × 10^7^), followed by F₁ seeds (8.60 × 10^4^–1.40 × 10^6^) and the lowest in cowpea seeds (3.19 × 10^4^–1.36 × 10^5^) harvested from mechanically sap inoculated plants. CPMMV infection significantly reduced seed quality parameters including 100-seed weight (4.32–6.66gm), germination percentage (55–81%), fresh weight and seedling vigor indices I and II in both the soybean genotypes. Biochemical analysis showed a marked elevation in H₂O₂, PAL and CAT activity while POX, SOD and total phenol content showed non-significant increases. Collectively, this study provides the definitive evidence of seed transmission of the CPMMV-VNB isolate in soybean and cowpea, highlighting its detrimental effects on seed quality and reinforcing its epidemiological importance in legume pathology.

## 1 Introduction

Soybean (*Glycine max*) is a globally important leguminous crop, valued for its high protein content, oil production, and broad industrial utility. It plays a pivotal role in ensuring food and nutritional security, serving as a primary source of plant-based protein for both human diets and animal feed (Hartman et al., 2011; Kumari et al., 2025). However, soybean production is increasingly threatened by diverse biotic and abiotic stresses. Among these, plant viruses are particularly detrimental, causing considerable yield and quality losses across major cropping systems and contributing to significant global economic losses annually (Jones, 2021). Despite the progress in agronomic practices and resistance breeding, viral pathogens continue to pose a persistent challenge to soybean cultivation. Notably, viruses belonging to at least 22 genera have been reported to naturally infect soybean under field conditions worldwide, highlighting the need for improved surveillance, diagnostics, and integrated disease management strategies (Leastro et al., 2024).

Seed transmission serves as a critical mechanism for the long-term persistence of plant viruses, particularly in the absence of suitable hosts or vectors. Many seed-transmitted viruses are capable of surviving within viable seeds for extended periods, thereby facilitating their continued presence across growing seasons (Sastry, 2013). Epidemiologically, seed transmission functions as a major source of primary inoculum for vertically transmitted viruses, which can subsequently be disseminated by insect vectors (Dwyer et al., 2007). In the context of a changing climate, this transmission route offers substantial advantages to viruses by enabling survival under unfavorable environmental conditions, thus promoting their spread into new geographic regions (Jones, 2016). Current estimates suggest that approximately 25% of described plant viruses are transmitted through seeds, and it has been projected that up to one-third of all plant viruses may ultimately be identified as seed-transmissible in at least one host species (Baldodiya et al., 2020; Gil-Valle et al., 2024).

Seed-transmitted viruses can reach developing seeds through either direct invasion of embryonic tissues or indirect invasion of reproductive structures (Pagán, 2022; Sandra and Mandal, 2024). Additionally, some plant viruses may be transmitted externally via seed surfaces, where virus particles adhere to the seed coat without penetrating the embryo (Hull, 2014; Isogai et al., 2015). However, establishment of host pathogen interactions in the embryo is generally essential for successful internal seed transmission (Mink, 1993; Sastry, 2013; Sandra and Mandal, 2024). During the process of embryo invasion, viruses can result in detrimental morphological and genetic alterations, contributing to significant yield and economic losses (Escalante et al., 2024).

Recently from the last decade veinal necrosis, leaf blight, stunting and bud blight symptoms become severe in some of the soybean genotypes which ultimately lead to collapse of the seedlings under Indian conditions. A diverse group of viruses were known to cause necrotic symptoms in soybean *viz.,* alfalfa mosaic virus (AMV, *Bromoviridae*), bean pod mottle virus (BPMV, *Secoviridae*), cowpea mild mottle virus (CPMMV, *Betaflexiviridae*), groundnut bud necrosis virus (GBNV, *Tospoviridae*), soybean mosaic virus (SMV, *Potyviridae*), soybean vein necrosis virus (SVNV, *Potyviridae*), tobacco ringspot virus (TRSV, *Secoviridae*) (Bhat et al., 2002; Zanardo and Carvalho, 2017; Hameed et al., 2022) and tobacco streak virus (TSV, *Bromoviridae*) (Arunkumar et al., 2008). In our initial studies, soybean veinal necrosis and bud blight (VNB) disease was found to be caused by CPMMV but not the other viruses.

CPMMV belongs to the genus *Carlavirus* of *Betaflexiviridae* family with positive sense ss-RNA of flexuous filamentous particle (10–15 × 610–700 nm). The viral genome consists of 8,200 nucleotides with 5′ cap structure (m7GpppG) and 3′ poly (A) tail encoding six open reading frames (ORFs) (Menzel et al., 2010; Zanardo et al., 2014). CPMMV was initially documented in Ghana in 1973 (Brunt and Kenten, 1973), and in India it was first reported in groundnut (*Arachis hypogaea*) in 1984 (Iizuka et al., 1984). In recent years, CPMMV has been reported as an emerging threat to soybean crops with disease incidence in affected fields ranging from 10 to 85% (Yadav et al., 2023). There were extensive studies on transmission of CPMMV through whiteflies (*Bemisia tabaci*) in a non-persistent manner (Iwaki et al., 1982; Muniyappa and Reddy, 1983; Jeyanandarajah and Brunt, 1993; Naidu et al., 1998; Marubayashi et al., 2010). However, scanty information is available regarding seed transmission and influence on seed quality.

Seed quality is crucial for successful germination, seedling vigor, plant establishment, yield potential and resistance to pathogens in soybean. Good quality seed can ensure uniform crop stand whereas disease infected seeds can reduce productivity, and have an adverse economic impact associated with reduced market value and levels of oil and protein (Hu et al., 2023). Hence, understanding the impact of viral infections on seed quality traits is necessary for developing effective disease management strategies and sustaining global soybean production. So the present study was undertaken to investigate the seed transmissibility of CPMMV-VNB isolate in soybean. The investigation included of two experimental aspects: assessment of transmission from naturally infected soybean seeds and from cowpea (*Vigna unguiculata*) plants mechanically inoculated with CPMMV. In addition, the effect of viral infection on key seed quality parameters and enzymatic activities was also evaluated to provide comprehensive understanding of the biological and physiological consequences of CPMMV-VNB infection in soybean.

## 2 Materials and methods

### 2.1 Source of virus and plant material

A systematic field survey was carried out during the *kharif* season of 2024–25 at the experimental fields of the Indian Agricultural Research Institute (IARI), New Delhi, to investigate the seed transmissibility of the virus associated with veinal necrosis and bud blight (VNB) disease in soybean. Soybean plants exhibiting characteristic symptoms of veinal necrosis, foliar blight and bud blight were tagged at 25 days after sowing (DAS). The dried pods were harvested from the survived symptomatic plants; seed was separated, labeled and stored at optimal moisture content for further studies. The seed was primarily collected from two soybean genotypes *viz.,* Asb-114 and AMS-2022-1 and pooled together due to less number of pods produced.

The cowpea genotypes Arka Samrudhi and Arka Suman (Obtained from IIHR, Bengaluru, Karnataka) were also mechanically sap inoculated with soybean leaf tissue collected from VNB diseased plants. For mechanical sap inoculation, the soybean leaf tissue macerated in 0.1 M phosphate buffer (pH 7.0) with 0.1% *β*-mercaptoethanol and pinch of celite. Ten seedlings of each cowpea genotype, in two replicates mechanically inoculated after dusting with 600-mesh carborundum. Post inoculation, plants were maintained under controlled environmental conditions in the National Phytotron Facility (NPF), IARI, New Delhi, at 26 ± 2 °C with a 16/8 h light/dark photoperiod. Upon maturation, pods were harvested from the infected plants; seed was separated and stored for further studies.

### 2.2 Serological detection of CPMMV-VNB isolate from seed material

To confirm the presence of CPMMV in seed samples, a direct antigen coated enzyme linked immunosorbent assay (DAC-ELISA) was employed using CPMMV specific polyclonal antibodies (1:200; Nano Diagnostics, USA). Seeds were initially surface sterilized using 1% sodium hypochlorite for 1 min (Sauer and Burroughs, 1986; Sandra et al., 2020a), followed by two rinses with sterile distilled water and subsequent imbibition in water for 8–10 h. To standardize the number of seed required for reliable detection, seed extracts were prepared by grinding whole seeds in groups of one, three, and five, using coating buffer (1:1 w/v) supplemented with 2% polyvinylpyrrolidone (MW 40,000) (Kothandaraman et al., 2016). 200 μL of seed extract was dispensed into 96-well polystyrene microtiter plates (Corning, Sigma, USA) and incubated at 37 °C for 1 h. After washing the plates three times with PBS-T (phosphate-buffered saline containing Tween-20), 200 μL of blocking solution (1% w/v bovine serum albumin in PBS-T) was added and incubation was done at 37 °C for 1 h. After another set of washes, a 1:200 dilution of CPMMV polyclonal antibodies was added, and plates were incubated at 37 °C for 1 h. Subsequently, a goat anti-rabbit IgG alkaline phosphatase conjugate (1,20,000; Sigma, USA) was added and the plate was incubated for 1 h at 37 °C. After a final wash, 0.5 mg/mL of *p*-nitrophenyl phosphate (Sigma, USA) was added as the substrate, and the plates were incubated in the dark at 37 °C for 1 h. Optical density was measured using an ELISA plate reader at 405 nm (BIO-TEK Instruments, USA).

The seed transmission nature of CPMMV-VNB isolate was also evaluated in mechanically sap inoculated cowpea genotypes Arka Samrudhi and Arka Suman using DAC-ELISA in the group of 3 whole seeds. Samples exhibiting absorbance values at least twice that of healthy control were considered positive for CPMMV infection.

### 2.3 Total RNA isolation and reverse transcription polymerase chain reaction (RT-PCR)

Prior to total RNA extraction, starch was eliminated from soybean seeds through starch removal extraction buffer comprising 1 M Tris–HCl (pH 8.0), 5 M LiCl, 0.5 M EDTA (pH 8.0) and 5% SDS (Li and Trick, 2005). The aqueous phase obtained following starch removal was used for RNA extraction using the SV Total RNA Isolation System (Promega, Madison, USA). Total RNA was isolated from single seeds, group of three and five whole seed, seed coat, cotyledons, and embryonic axis. RNA was quantified spectrophotometrically using the NanoDrop™ 2000 (Thermo Fisher Scientific, India). First-strand cDNA synthesis was carried out using the PrimeScript™ cDNA synthesis kit (TaKaRa, Japan) in a 20 μL reaction volume comprising 4.0 μL of 5 × PrimeScript buffer, 1.0 μL of 10 mM dNTP mix, 1.0 μL of reverse transcriptase, 0.5 μL of RNase inhibitor, 1.0 μL of 10 mM reverse primer (NS77R), and 1–5 μL of total RNA (equivalent to 500 ng). The reaction consisted of incubation at 30 °C for 10 min, followed by 42 °C for 60 min, and finally at 70 °C for 15 min using a thermal cycler. The PCR was conducted in a 25 μL reaction mixture containing 1.0 μL of synthesized cDNA, 2.5 μL of 10 × reaction buffer, 2.5 μL of 2 mM dNTP mix, 1.0 μL each of forward and reverse CPMMV-CP specific primers (NS77F and NS77R; Table 1), 0.2 μL of DreamTaq DNA polymerase (5 U/μl), and nuclease free water to adjust the volume. The PCR programme included: initial denaturation at 95 °C for 30 s, primer annealing at 55 °C for 40 s and extension at 72 °C for 1 min per kilobase, and a final extension at 72 °C for 10 min. PCR products were resolved on 1.2% agarose gels using 1 kb DNA ladder and visualized under UV transilluminator. The amplified CP specific band from the whole seed sample was excised and purified using the Wizard® SV Gel and PCR Clean-Up System (Promega, Madison, WI, USA). The ligation of purified PCR product was done in the pDrive cloning vector followed by transformation in *E. coli* DHα competent cells. Recombinant clones were confirmed through colony PCR, restriction digestion and subsequently sequenced using the Sanger dideoxy method.

**Table 1:** List of primers designed for the amplification and quantification of CPMMV-VNB isolate from various seed tissues.

S. N	Primer	Primer sequence (5′ to 3′)	Expected product (bp)	Annealing temperature (°C)	Region of the genome amplified
1	NS77F	atggagtcwgtrtttgatttaaa	867 bp	55	Complete CP region
	NS77R	ytacttcttggcgtgattgaaatt			
2	NS180F	gattggaagggtggttctatac	149 bp	58	Partial CP region for RT-qPCR
	NS180R	atagctgcccaatcagaaggtg			

Total RNA was also extracted from systemic leaves of cowpea genotypes that had been mechanically sap inoculated. Total RNA was also extracted from five whole seeds, seed coats, cotyledons, and embryonic axis after starch removal. The extracted RNA was subsequently used for first strand cDNA synthesis and PCR amplification using CPMMV CP specific primers as described above (Table 1).

### 2.4 Viral load determination through quantitative RT-PCR

To quantify CPMMV viral RNA titers in field infected soybean seed material and seeds derived from mechanically sap inoculated cowpea genotypes, reverse transcription quantitative PCR (RT-qPCR) was employed. 1.0 μL of cDNA synthesized from 500 ng of total RNA using a virus-specific reverse primer (NS77R), served as the template for this purpose. Quantification was performed on group of five whole seed, seed coats, cotyledons, and embryonic axis from both soybean and cowpea using the primers NS180F and NS181R (Table 1) hybridizing to CP region using TB Green® Premix Ex Taq™ II (Tli RNaseH Plus) (TaKaRa, Japan). Primer specificity was confirmed via conventional PCR prior to RT-qPCR analysis. Each biological replicate included three technical replicates per reaction. The RT-qPCR thermal cycling programme beginning with an initial denaturation at 95 °C for 1 min, then went through 40 cycles of 95 °C for 15 s, 58 °C for 30s and 72 °C for 5 s. Amplification reactions were conducted using the Mx3005P QPCR system (Agilent Technologies, India). A standard curve was generated from tenfold serial dilutions of a plasmid DNA construct harboring the CPMMV CP gene to determine absolute viral copy numbers. Quantification of viral load in various seed components was performed using standard formulas (Supplementary material).

### 2.5 Seed transmission efficiency of CPMMV

The seed transmission efficiency of CPMMV-VNB infected soybean seed was evaluated through grow-out tests or progeny assay. To conduct the grow out test, 100 seeds each from symptomatic and asymptomatic soybean plants were potted separately and kept in glasshouses at NPF, IARI. The plants were observed for the onset of symptoms at regular intervals upto 40 DAS and symptoms were documented. To determine the presence of CPMMV, first trifoliate leaves of germinated seedlings were checked through DAC-ELISA using CPMMV polyclonal antibodies at 10–15 DAS. Total RNA was extracted for RT-PCR from second trifoliate leaves at 15–20 DAS using SV total RNA isolation system and with CP specific primers NS77F and NS77R, RT-PCR was performed. Viral load was quantified through RT-qPCR in the germinated seedlings as described in previous section. To observe the vertical transmission of CPMMV via seed in F2 generation, the seed obtained from F1 generation plants was sown. The presence of CPMMV was assessed in the F2 generation seedlings using polyclonal antibodies through DAC-ELISA and RT-PCR with CP specific primers.

### 2.6 Effect of CPMMV on seed quality parameters

To assess the influence of CPMMV on seed quality, observations on parameters such as 100 seed weight, germination %, seedling vigor index I and II were made. Germination % was calculated by putting the 25 seeds from both infected and healthy plants in four replications using between paper (BP) method in accordance with International Seed Testing Association Standards (ISTA, 2024). The first and final counts for germination were noted at 5 and 8 days, respectively. Germination percentage was calculated by considering only normal seedlings. The seedling length (root length + shoot length) of healthy and infected seedlings was measured in centimetres to calculate vigor index I. To determine vigor index II, ten seedlings from each replication with measured seedling length were dried at 120 °C for 24 h before being weighed (Abdul-Baki and Anderson, 1973; Supplementary material).

### 2.7 Effect of CPMMV on soybean seed biochemical system infected with VNB disease

To evaluate the effect of CPMMV on the antioxidant enzymes, the leaf tissue of the germinated seedlings collected on final count day was used for catalase (CAT), peroxidase (POX) and superoxide dismutase (SOD) estimation. Enzyme extraction was performed by homogenizing 0.5 g of leaf tissue in 5 mL of chilled 50 mM potassium phosphate buffer (pH 7.0) containing 1 mM EDTA and 1% (w/v) polyvinylpyrrolidone (PVP). The centrifugation of homogenate was done at 12,000 rpm for 15 min at 4 °C, and the resulting supernatant was used for enzyme assays.

#### 2.7.1 Catalase activity assay

The CAT activity was determined based on the rate of H₂O₂ decomposition following the method proposed by Aebi (1984). Briefly, the reaction was initiated following addition of 1 mL of 15 mM H₂O₂ to 50 μL of the crude enzyme extract, 950 μL of double distilled water (DDW) and 1 mL of 50 mM phosphate buffer (pH 7.0) (total 3.0 mL). The CAT activity was determined based on the rate of H_2_O_2_ decomposition measured in the spectrophotometer at 240 nm for 3 min at 30 s interval at 25 °C. An extinction coefficient of 39.4 mM^−1^ cm^−1^ was used to calculate CAT activity and expressed as μmol of H₂O₂ decomposed per minute per gram fresh weight (μmol H₂O₂ decomposed/min/g FW).

#### 2.7.2 Peroxidase activity assay

The POX activity was assayed following the method of Chance and Maehly (1955). The reaction mixture consisted of 1 mL of 50 mM phosphate buffer (pH 7.0), 0.5 mL of 20 mM guaiacol, 0.5 mL of 20 mM hydrogen peroxide (H₂O₂), 50 μL of enzyme extract and 950 μL of DDW. Initiation of reaction was done by the addition of H₂O₂, and the increase in absorbance was recorded at 470 nm for 3 min due to the formation of tetraguaiacol. The change in absorbance per minute was used to evaluate enzyme activity using the extinction coefficient of 26.6 mM^−1^ cm^−1^ for tetraguaiacol. Results were expressed as μmol of tetraguaiacol formed per minute per gram fresh weight (μmol tetraguaiacol formed/min/g FW).

#### 2.7.3 Superoxide dismutase activity assay

The SOD activity was assayed using the method described by Giannopolitis and Ries (1977), which measures the capacity of SOD to photochemically reduce the nitroblue tetrazolium (NBT) in the reaction solution. The reaction mixture (3.0 mL) consisted of 1 mL of 50 mM phosphate buffer (pH 7.8), 0.2 mL of 13 mM methionine, 0.2 mL of 75 μM NBT, 0.2 mL of 2 μM riboflavin, 0.2 mL of 100 μM EDTA, 50 μL of enzyme extract and 950 μL of DDW to make up volume. The reaction was carried out under fluorescent light for 10 min. The reaction mixture was kept in darkness for 10 min for the control samples. Following exposure to light, the light was switched off and the production of a blue colored compound formazan, formed from the photoreduction of NTB and the control samples were measured by recording the absorbance at 560 nm. To calculate the SOD activity, the values from the experimental samples (light) were deducted from the values from the control samples. One unit of SOD activity was defined as the amount of enzyme required to cause 50% inhibition of NBT reduction under assay conditions and was expressed as units per gram fresh weight (Units/g FW) (Beauchamp and Fridovich, 1971).

#### 2.7.4 Phenylalanine ammonia lyase (PAL) activity assay

The PAL activity was determined according to the method of Ross and Sederoff (1992) with slight modifications. 0.5 g of fresh leaf tissue was homogenized in 1 mL of 0.1 M sodium borate buffer (pH 8.8), centrifuged at 15,000 rpm for 20 min at 4 °C and resultant supernatant was used as the enzyme extract. The assay mixture (total volume 1.5 mL) contained 0.5 mL of 0.1 M borate buffer (pH 8.8), 0.5 mL of 10 mM L-phenylalanine (substrate), and 0.5 mL of enzyme extract. The reaction was incubated at 30 °C for 30 min, and the formation of trans-cinnamic acid was noted by taking the absorbance at 290 nm. A control reaction without substrate was run in parallel to correct for background absorbance. The molar extinction coefficient of trans-cinnamic acid (*ε* = 9,630 M^−1^ cm^−1^ at 290 nm) was used for calculation of enzyme activity, which was expressed as nmol trans-cinnamic acid produced per minute per gram fresh weight (nmol of TCA formed/min/g FW).

#### 2.7.5 Total phenol content

Total phenolic content was estimated using the Folin-Ciocalteu method as described by Singleton and Rossi (1965), with minor modifications. Fresh leaf tissue (0.5 g) was homogenized in 5 mL of 80% ethanol, centrifuged at 10,000 rpm for 30 min at 4 °C, and the supernatant was collected for analysis. An aliquot of 0.5 mL of the ethanolic extract was mixed with 2.5 mL of 10% Folin–Ciocalteu reagent (diluted with distilled water) and incubated for 5 min. Then, 2.0 mL of 7.5% (w/v) sodium carbonate (Na₂CO₃) solution was added, and the reaction mixture was incubated at 45 °C in the dark for 45 min. The absorbance of the resulting blue color was measured at 765 nm using a UV–Vis spectrophotometer. A standard calibration curve was prepared using gallic acid (0–100 μg mL^−1^), and the total phenolic content was expressed as mg gallic acid equivalents (GAE) per gram fresh weight (mg GAE/g FW).

#### 2.7.6 Determination of hydrogen peroxide (H_2_O_2_)

Hydrogen peroxide content was quantified following the method of Velikova et al. (2000) with slight modifications. 0.5 g of fresh leaf tissue was ground in 5 mL of ice cold 0.1% (w/v) trichloroacetic acid in a pre-chilled mortar and pestle. The homogenate centrifugation was done at 12,000 × g for 15 min at 4 °C. To 0.5 mL of the supernatant, 0.5 mL of 10 mM potassium phosphate buffer (pH 7.0) and 1 mL of 1 M potassium iodide (KI) were added. The reaction mixture was incubated in the dark for 10 min at room temperature. The absorbance of the titanium–hydroperoxide complex was measured at 390 nm using a UV–Vis spectrophotometer. A standard curve was prepared using known concentrations of hydrogen peroxide (0–100 μmol), and H₂O₂ content in the samples was expressed as μmol H₂O₂ per gram fresh weight (μmol/g FW).

### 2.8 Statistical analysis

Statistical analysis was performed using R Studio (version 2024.12.0 + 467) with rstatix package. To evaluate the effect of disease on seed quality, a paired *t*-test (Ruxton, 2006) was applied to compare values between healthy and diseased seed samples for each genotype (Asb-114 and AMS-2022-1). Statistical significance was considered at *p* < 0.05. Results were presented as mean ± standard deviation (SD) along with corresponding *t* and *p* values.

## 3 Results

### 3.1 Identification of CPMMV location in seeds produced from infected soybean and cowpea plants

**Figure 1:**
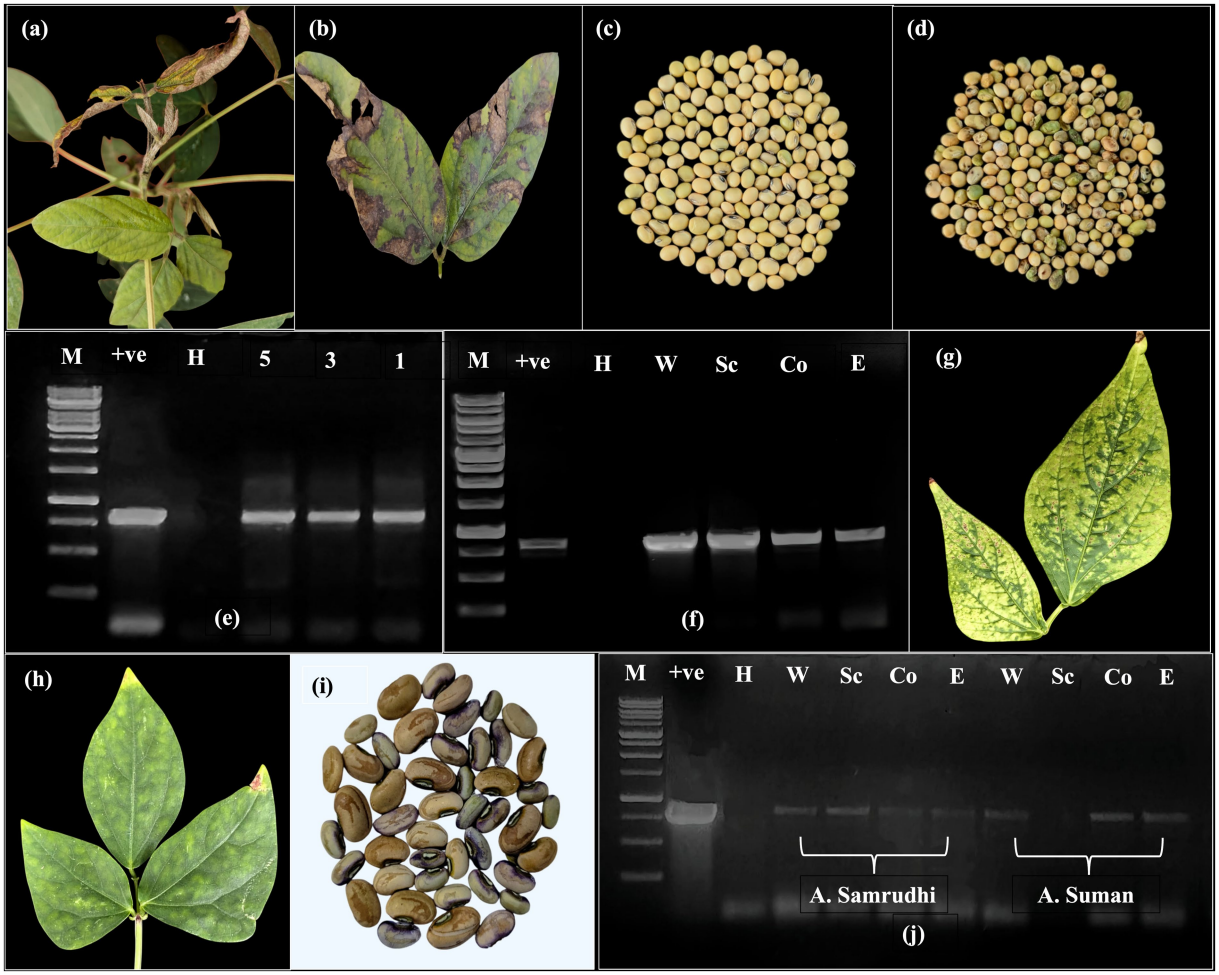
Confirmation of seed transmissibility in soybean and cowpea. **(a)** Symptomatic soybean plant showing necrosis and bud blight. **(b)** Necrotic symptoms on the leaf. **(c)** Healthy seed harvested from non-symptomatic plant. **(d)** Diseased seed harvested from necrotic CPMMV infected plant showing crinkling and discolouration. **(e)** Detection of CPMMV through RT-PCR with CP specific primers in group of five, three and single seed (H-RT-PCR from healthy whole seed; +ve-Leaf tissue infected with CPMMV; M-Generuler 1 kb DNA ladder) **(f)** PCR amplicons (867 bp) obtained with CP specific primers NS77F and NS77R in different seed parts (W-Whole seed; Sc-Seed coat; Co-Cotyledons; E-Embryo). **(g)** Symptoms of chlorotic and necrotic spots sap inoculated Arka Suman. **(h)** Chlorotic spots and blotches Arka Samrudhi. (i) Discolored and low weight seeds obtained from mechanically sap inoculated cowpea plants. **(j)** RT-PCR confirmation of CPMMV in whole seed and seed parts of mechanically sap inoculated cowpea cvs A. Samrudhi and A. Suman.

Soybean plants displayed the symptoms of veinal necrosis, chlorosis, mottling, and bud blight were found to be mainly due to CPMMV in previous studies (Figures 1a,b). Most of the soybean plants showing VNB symptoms were died by 40 DAS. The seed collected from the survived plants was reduced in size, malformed with seed coat discolouration (Figures 1c,d). DAC-ELISA testing of soybean seed in the group of five whole seed, seed coat, cotyledon and embryo axis showed the absorbance values of 0.275–0.347 which were at par with healthy tissue and become inconclusive for virus detection. The RT-PCR using CPMMV-CP specific primers successfully amplified an 867 bp fragment from single seed, group of three and five seeds (Figure 1e). Further, a consistent amplification was observed in dissected seed parts including the seed coat, cotyledons, and embryo from groups of five seeds (Figure 1f). Sequencing of the amplified product from whole seeds revealed 97.68% nucleotide identity with a CPMMV isolate from urdbean (*Vigna unguiculata*) in India (GenBank: MH345698), and 86.95 and 86.84% identity with CPMMV soybean isolates from China (MW354943) and Brazil (PQ834430), respectively.

Mechanical sap inoculation of cowpea genotypes Arka Samrudhi and Arka Suman led to the development of localized symptoms at 15–18 days post-inoculation (dpi), followed by systemic symptoms at 23–28 dpi. The most frequently observed symptoms included chlorotic spots and blotches in Arka Samrudhi, with an infection rate of 60%, and necrotic and chlorotic spots in Arka Suman, with an infection rate of 45% (Figures 1g,h). No symptoms were observed in the mock-inoculated control plants. Seeds were harvested from plants confirmed to be CPMMV-positive by RT-PCR. Dry seeds from tagged, infected plants showed varying phenotypes ranging from asymptomatic to visible seed coat discoloration, along with reduced seed weight (Figure 1i). RT-PCR with CPMMV CP specific primers confirmed virus presence in all seed parts, i.e., whole seed, seed coat, cotyledon and embryo of both genotypes, with the exception of the seed coat in Arka Suman, where no amplification was detected (Figure 1j). These findings confirmed that mechanical sap inoculation of cowpea with CPMMV-VNB isolate was capable of resulting in systemic infection and vertical transmission through seed.

### 3.2 Seed transmission efficiency of CPMMV in the next generations

**Figure 2:**
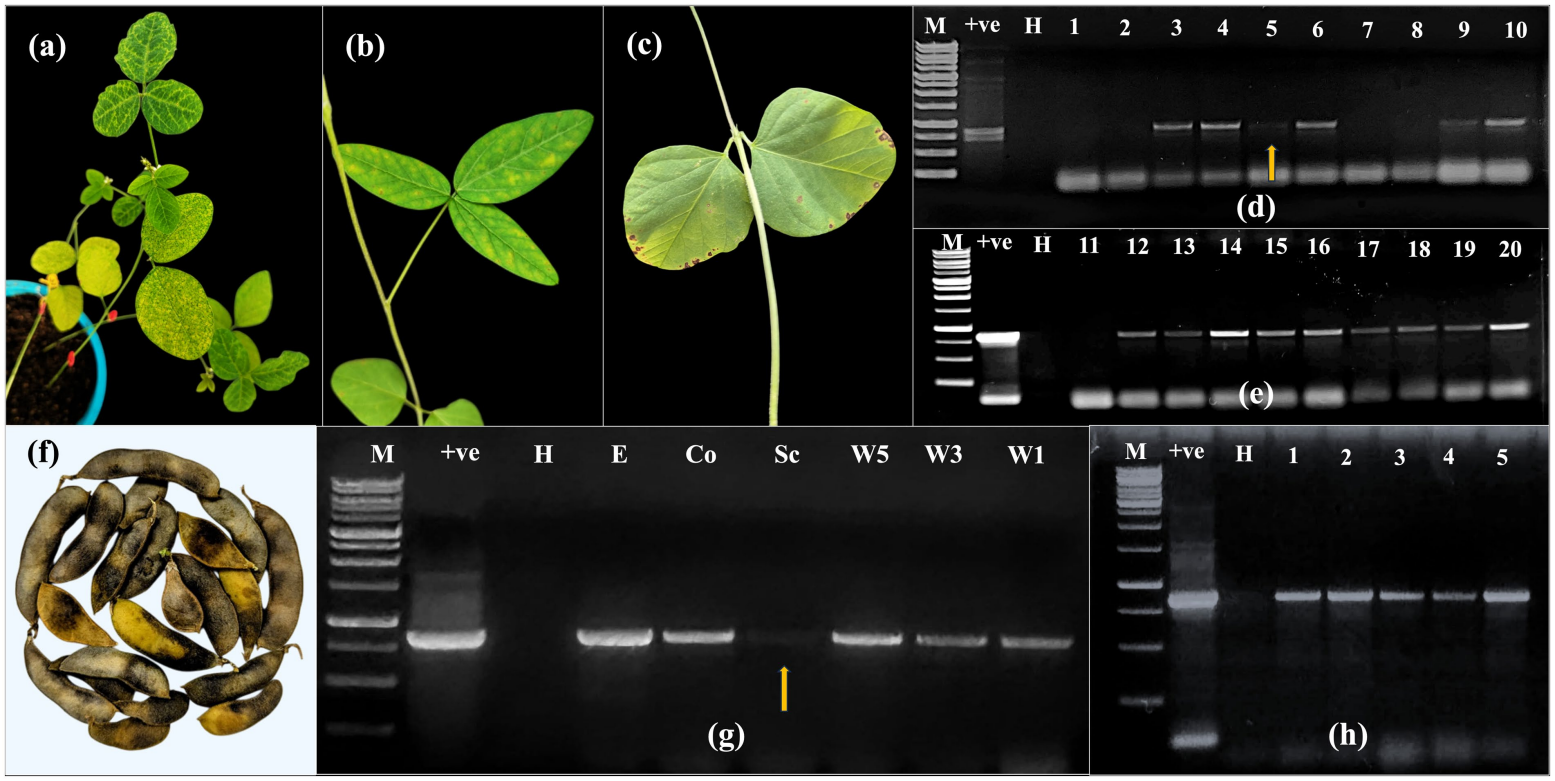
Confirmation of seed transmission efficiency of CPMMV in the further generations. **(a,b)** Symptoms of chlorotic spots, chlorotic blotches and necrotic spots in grow out test F1 generation plants. **(c)** Appearance of symptomatic necrotic spots in the F2 generation plants grown from seeds harvested from F1 CPMMV infected plants. **(d,e)** Detection of CPMMV with RT-PCR in seedlings of 20 plants of F1 generation, yielding the 867 bp amplicon with CP specific primers (H-RT-PCR from healthy whole seed/plant; +ve-Leaf tissue infected with CPMMV; M-Generuler 1 kb DNA ladder) **(f)** Pods displaying necrotic spots harvested from the F1 plants. **(g)** RT-PCR confirmation for presence of CPMMV in whole seeds and seed parts harvested from F1 plants with CP specific primers (W5, W3 and W1-Whole seed in the group of 5 seeds, 3 seeds and 1 seed respectively; Sc-Seed coat; Co-Cotyledons; E-Embryo). **(h)** Detection of CPMMV through RT-PCR with CP specific primers in five F2 plants grown from the F1 seed. (Light amplification is shown with arrow).

Seed harvested from CPMMV infected soybean plants were evaluated for seed transmissibility through a grow out test. Germination of infected seeds was delayed, initiating around 8–10 DAS. Of the 100 seeds sown, 66 germinated, resulting in a germination percentage of 66%. The emerged seedlings exhibited stunted growth and early symptoms such as mild chlorotic and necrotic spots. As growth progressed, symptoms intensified to include prominent necrotic spots, chlorotic blotches, and leaf puckering in several plants (Figures 2a,b). Among the 66 seedlings, visible symptoms were observed in 30 plants. DAC-ELISA performed on 50 randomly selected seedlings using CPMMV polyclonal antibodies showed the negative results. RT-PCR performed using CPMMV CP specific primers on 20 symptomatic soybean seedlings in F1 generation showed the 867 bp amplicon from 15 plants and confirmed the presence of CPMMV (Figures 2d,e). These findings clearly demonstrate that CPMMV is seed transmissible in soybean with a 75% of seed to seedling transmission efficiency.

Grow-out test was also conducted to evaluate the vertical transmission of CPMMV to the F2 generation using the seed harvested from F1 generation soybean plants, previously confirmed for the presence of CPMMV. A very limited number of pods were developed in the F1 plants and the seed harvested from these pods was discolored. The RT-PCR conducted on soybean seed harvested from F1 plants using CPMMV CP specific primers resulted in the consistent amplification of 867 bp from single, group of three, and five whole seed as well as in dissected embryonic axis, cotyledons and seed coat tissue (Figure 2g). For the grow-out test, 20 F2 seeds were sown, out of which 11 seeds germinated (55% germination). The seedlings in F3 generation also exhibited necrotic and chlorotic spots (Figure 2c). The germinated seedlings tested through DAC-ELISA with CPMMV polyclonal antibodies reacted negatively. Interestingly, RT-PCR analysis of five randomly selected symptomatic seedlings, with CPMMV specific primers showed the characteristic 867 bp CP specific amplicon (Figure 2h). These findings suggested a 100% seed transmission rate in the tested F2 seed samples, confirming vertical transmission of CPMMV-VNB isolate to the third generation.

### 3.3 RT-qPCR analysis of CPMMV accumulation in infected seed tissues

The accumulation of CPMMV genomic RNA in seed tissues was quantified using RT-qPCR from naturally infected soybean seeds, seeds harvested from mechanically sap inoculated cowpea plants, and F1 seeds derived from grow-out tests of infected soybean plants. In field infected soybean seeds, CPMMV RNA was detected in all seed tissue components, with the highest viral load recorded in whole seed (5.35 × 10^7^ copies) and the lowest in the embryo (9.83 × 10^4^ copies) (Table 2). F1 seeds harvested from the grown seedlings exhibited moderate viral RNA accumulation, with whole seeds showing the highest accumulation (1.40 × 10^6^ copies) and lowest in seed coat (8.60 × 10^4^ copies). The seed harvested from the mechanically sap inoculated cowpea genotypes, Arka Samrudhi and Arka Suman showed viral RNA accumulation at par through RT-qPCR (Table 2). These results indicated that viral load in the field infected soybean seeds were several orders of magnitude higher than in the mechanically sap inoculated cowpea seeds. However, the titer value has reduced in subsequent F1 generation than the field infected soybean seeds. Nonetheless, the RT-qPCR data clearly confirmed that CPMMV-VNB can be transmitted through seeds in both naturally infected soybean and mechanically sap inoculated cowpea genotypes.

**Table 2:** Quantification of viral RNA from infected seed tissues of soybean and cowpea through quantitative real time reverse transcription polymerase chain reaction (RT-qPCR).

Seed part	Ct Value (mean ± standard error)	Copy number
Field infected soybean seed		
Whole seed	17.70 ± 0.04	5.35 × 10^7^
Seed coat	24.66 ± 0.24	4.25 × 10^5^
Cotyledons	20.73 ± 0.01	6.53 × 10^6^
Embryo	26.77 ± 0.11	9.83 × 10^4^
F1 soybean seed		
Whole seed	22.94 ± 0.30	1.40 × 10^6^
Seed coat	26.96 ± 0.08	8.60 × 10^4^
Cotyledons	25.71 ± 0.32	2.05 × 10^5^
Embryo	26.54 ± 0.23	1.15 × 10^5^
Seed harvested from mechanically sap inoculated cowpea genotypes		
Arka Samrudhi		
Whole seed	26.56 ± 0.19	1.14 × 10^5^
Seed coat	26.57 ± 0.38	1.13 × 10^5^
Cotyledons	27.36 ± 0.63	6.51 × 10^4^
Embryo	28.12 ± 0.14	3.84 × 10^4^
Arka Suman		
Whole seed	28.01 ± 0.32	4.14 × 10^4^
Seed coat	28.39 ± 0.44	3.19 × 10^4^
Cotyledons	27.09 ± 0.37	7.89 × 10^4^
Embryo	26.30 ± 0.22	1.36 × 10^5^

### 3.4 Effect of CPMMV infection on seed quality parameters

**Figure 3:**
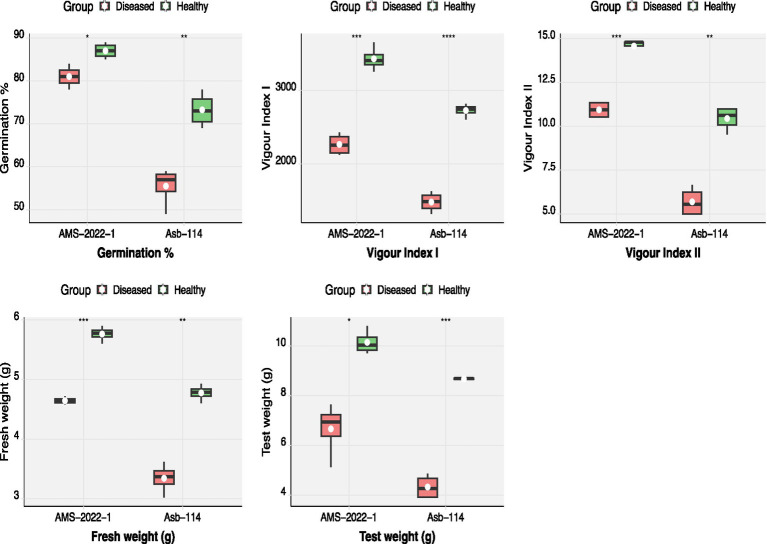
Effect of CPMMV virus infection on seed quality parameters *viz.,* germination percentage, fresh weight, test weight, vigor index I and II (Significance levels: *p ≤ 0.05; **p ≤ 0.01; *** *p* ≤ 0.001; **** *p* ≤ 0.0001).

Seed quality parameters were comprehensively evaluated in two soybean genotypes, Asb-114 and AMS-2022-1, which tested positive for CPMMV via DAC-ELISA and RT-PCR. Virus infection was found to be significantly affect all assessed seed quality traits in both genotypes (Figure 3; Table 3). In Asb-114, CPMMV infection caused a substantial reduction in germination percentage, which declined from 73.25% in healthy seeds to 55.50% in infected seeds. Similarly, in AMS-2022-1, germination dropped from 87.00% in healthy seeds to 81.00% in infected seeds, indicating a genotype-dependent variation in the impact of the virus on seed viability. Seedling vigor index-I which is a function of seedling length and germination %, was markedly lower in seeds from infected plants of both genotypes highlighting the negative effect of CPMMV on early seedling growth and establishment. Seedling vigor index II, which reflects seedling dry biomass accumulation, also showed a drastic decline in the infected seeds, as both fresh and dry weights of seedlings were consistently lower compared to their healthy counterparts (Table 3). The 100-seed weight (test weight), a key indicator of seed development and filling, was significantly reduced in the infected plants. In Asb-114, the test weight dropped from 8.67 g in healthy seeds to 4.32 g in infected seeds. Similarly, AMS-2022-1 exhibited a decline from 10.14 g (healthy) to 6.66 g (infected) in the test weight. Collectively, these findings demonstrated that CPMMV infection substantially compromises seed quality in soybean by reducing germination potential, seedling vigor and seed weight.

**Table 3:** Effect of CPMMV virus infection on seed quality parameters.

Parameter	Genotype	Healthy Mean ± SD	Diseased Mean ± SD	*t*-value	*p*-value
Germination percentage	Asb-114	73.25 ± 4.03	55.50 ± 4.51	6.25	0.0083
	AMS-2022-1	87.00 ± 1.83	81.00 ± 2.58	4.08	0.0267
Vigor Index-I	Asb-114	2727.09 ± 91.75	1482.36 ± 138.54	20.82	0.0002
	AMS-2022-1	3429.32 ± 167.27	2265.36 ± 148.96	12.97	0.0010
Vigor Index-II	Asb-114	10.43 ± 0.70	5.68 ± 0.83	6.60	0.0071
	AMS-2022-1	14.57 ± 0.43	10.93 ± 0.46	17.48	0.0004
Fresh weight (g)	Asb-114	4.77 ± 0.13	3.34 ± 0.25	8.77	0.0031
	AMS-2022-1	5.76 ± 0.12	4.64 ± 0.05	20.97	0.0002
Test weight (g)	Asb-114	8.67 ± 0.04	4.32 ± 0.49	17.15	0.0004
	AMS-2022-1	10.14 ± 0.48	6.66 ± 1.08	4.58	0.0195

### 3.5 Effect of CPMMV virus infection on enzymatic parameters

**Figure 4:**
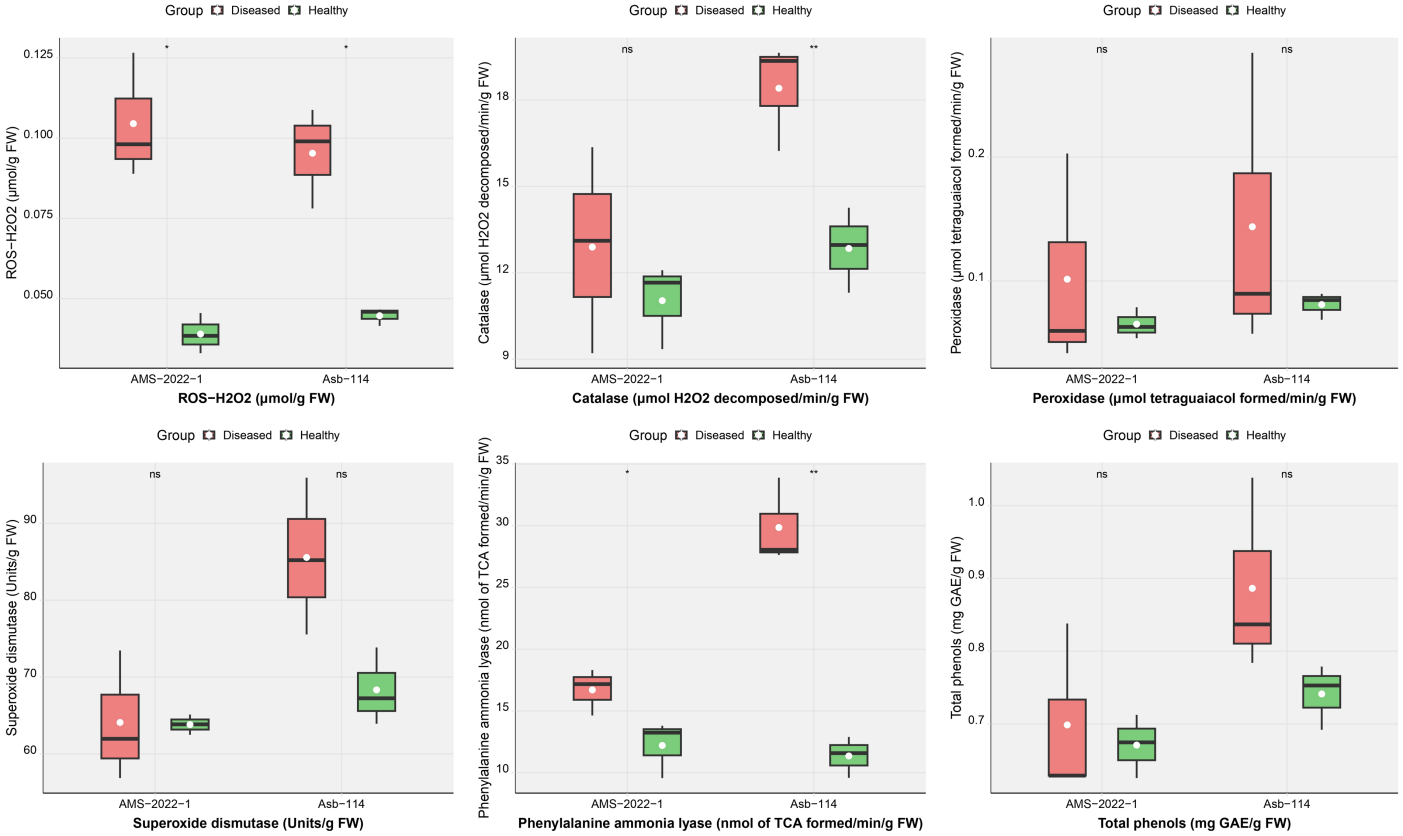
Effect of CPMMV virus infection on enzymatic parameters *viz*., H_2_O_2,_ Catalase (CAT), Peroxidase (POX), Superoxide dismutase (SOD), Phenylalanine Ammonia Lyase (PAL) and total phenols (Significance levels: *p ≤ 0.05; **p ≤ 0.01; ns = non-significant).

To understand the physiological stress and host defense responses triggered by seed-borne nature of CPMMV, key biochemical parameters were assessed in seedlings derived from both healthy and infected seeds of two soybean genotypes, Asb-114 and AMS-2022-1 (Figure 4; Table 4). The analyses focused on oxidative stress markers, antioxidant enzyme activities, and phenolic compounds, which are known to play critical roles in plant defense against biotic stress. H₂O₂ accumulation, a marker of oxidative stress and signaling molecule in plant-pathogen interactions, was significantly elevated in the diseased seedlings of both genotypes. Among antioxidant enzymes, CAT which catalyses the breakdown of H₂O₂ into water and oxygen, showed a significant increase in activity in infected seedlings of Asb-114, indicating an active enzymatic response to detoxify elevated ROS levels, while AMS-2022-1 exhibited only a marginal increase. POX and SOD two other key enzymes involved in ROS scavenging, showed a slight activity increase in the infected seedlings of both genotypes but with non-statistically significant figures. PAL, the main enzyme in the phenylpropanoid pathway associated with synthesis of defense related secondary metabolites, was significantly upregulated in infected seedlings of both genotypes. Total phenolic content, although consistently higher in infected seedlings compared to healthy controls across both genotypes, did not show statistically significant differences. Overall, the biochemical profiling revealed that CPMMV infection induced oxidative stress in soybean seedlings, with a genotype-dependent modulation of antioxidant and defense related enzyme systems, where Asb-114 showed a more pronounced antioxidant response compared to AMS-2022-1.

**Table 4:** Effect of CPMMV virus infection on enzymatic parameters.

Enzyme	Variety	Healthy Mean ± SD	Diseased Mean ± SD	*t*-value	*p*-value
Reactive Oxygen Species (H_2_O_2_) (μmol/g FW)	Asb-114	0.0446 ± 0.01	0.0953 ± 0.02	5.43	0.0322
	AMS-2022-1	0.0389 ± 0.01	0.1045 ± 0.02	4.67	0.0429
Catalase (μmol of H_2_O_2_ decomposed /min/g FW)	Asb-114	12.84 ± 1.48	18.41 ± 1.89	12.83	0.0060
	AMS-2022-1	11.03 ± 1.47	12.89 ± 3.58	0.78	0.5171
Peroxidase (μmol of tetraguaiacol formed /min/g FW)	Asb-114	0.0808 ± 0.01	0.1437 ± 0.12	0.81	0.5009
	AMS-2022-1	0.0650 ± 0.01	0.1073 ± 0.08	1.03	0.4110
Superoxide dismutase (units/g FW)	Asb-114	68.34 ± 5.05	85.56 ± 10.19	1.96	0.1881
	AMS-2022-1	63.82 ± 1.31	64.09 ± 8.50	0.04	0.9654
Phenylalanine ammonia lyase (nmol of transcinnamic acid formed/min/g FW)	Asb-114	11.35 ± 1.66	29.86 ± 3.49	13.94	0.0051
	AMS-2022-1	12.20 ± 2.30	16.70 ± 1.88	7.94	0.0155
Total phenols (mg GAE/g FW)	Asb-114	0.7411 ± 0.04	0.8864 ± 0.13	2.04	0.1771
	AMS-2022-1	0.6708 ± 0.04	0.6985 ± 0.12	0.54	0.6408

## 4 Discussion

Vertical transmission of plant viruses via seeds has been recognized for over a century and represents a critical mechanism for virus survival and dissemination between growing seasons (Doolittle, 1920; Johansen et al., 1994). Despite its significance, studies on seed transmission remain limited, primarily due to the challenges posed by typically low virus titers in seed tissues, which complicates detection, and the need for long duration, multi-generational experimentation. In our recent study, CPMMV was found to be associated with soybean veinal necrosis and bud blight disease leading to death of the plants under Indian conditions. As the symptoms appeared within 10–15 DAS, the seed transmission of CPMMV was suspected in soybean. Hence, a systematic study was carried out to understand the seed transmission nature of CPMMV necrotic isolate and disease influence on seed physiological and biochemical parameters.

The symptoms observed in CPMMV infected soybean plants, such as necrosis, chlorosis, mottling, and stunting, aligned with the previous reports of CPMMV infections (EPPO, 2022). Due to seedling mortality, there was no seed production, and plants that survived CPMMV infection produced fewer seeds than healthy controls. This might be due to the detrimental effects of viral infection on plant vigor and reproductive development (Song et al., 2016). When seed transmission is considered, detection of CPMMV in seed tissues alone is not a definitive indicator of its transmissibility. A considerable viral load is required to be detected through ELISA and RT-PCR, particularly in embryonic tissues, which is often necessary to facilitate vertical transmission (Pandey and Parmar, 2023; Ellis et al., 2020). When leaf tissues were analyzed using DAC-ELISA for CPMMV, it showed a positive reaction with CPMMV polyclonal antibodies. But, the harvested seed from CPMMV infected plants failed to produce positive results through DAC-ELISA. Similar discrepancy was reported in a study from Indonesia, where over 4,000 seeds collected from CPMMV infected soybean and groundnut plants tested negative via ELISA, even though the plants were confirmed infected (Horn et al., 1991). This inconsistency might be due to lower virus titers in seed tissues compared to vegetative tissues and also might be due to the virus strain used to develop polyclonal antibodies (Wei et al., 2022). The consistent amplification was observed from seed and seed parts in RT-PCR with CPMMV-CP specific primers, which might be due to higher sensitivity of RT-PCR, capable of detecting 1 pg. to 10 ng of target nucleic acid (Green and Sambrook, 2018). Similar findings have been reported in chili pepper mild mottle virus, where ELISA did not detect the virus in seeds, while RT-PCR using universal tobamovirus primers gave positive results (Ontañón et al., 2024). Hence, detecting CPMMV in soybean seed tissues with DAC-ELISA could not accurately predict seed transmissibility and should be used with caution to test seed for viral transmission. CPMMV was also found to be seed transmitted in cowpea and soybean genotypes through grow out tests and serological assays (Brunt and Kenten, 1973; Brito et al., 2012; Sutrawati et al., 2021). In contrast, few studies have reported the absence of seed transmission of CPMMV in soybean genotypes (Tavassoli et al., 2008).

In the experimental host cowpea, mechanical sap inoculation of CPMMV infected soybean leaves resulted in the necrotic symptoms and discolored seed material indicating the deleterious effect of viral infection on seed. The veinal necrotic symptoms were not observed in cowpea, as observed in field infected soybean, which might be due to species-specific symptom expression (Zanardo et al., 2014). However, DAC-ELISA could not detect the virus in both leaves and seeds which might be due to the lower sensitivity of polyclonal antibodies. RT-PCR analysis of systemic leaves, whole seeds and seed parts confirmed the presence of CPMMV through virus specific amplification. The detection of CPMMV in the embryo tissues of both cowpea genotypes, Arka Samrudhi and Arka Suman, indicated the capability of virus to get transmitted in next generation through seed. These findings were similar to previous studies on seed transmission of a soybean yellow mottle mosaic virus strain identified in India, where mechanical inoculation in French bean (*Phaseolus vulgaris*) resulted in confirmation of virus in seed embryos and subsequent transmission to the next generation (Sandra et al., 2020a). These results emphasized the potential of CPMMV to be vertically seed transmitted even in experimental hosts such as cowpea.

Usually progeny testing through grow out test is considered more accurate than direct seed testing for evaluating seed transmissibility of viruses, as it selectively detects the transmissible viruses and gets benefits due to greater viral titers in seedling tissues (Gil-Valle et al., 2024). In the present study, grow out tests with soybean seed harvested from VNB diseased plants confirmed the seed-to-seedling transmission of CPMMV, with a transmission rate of 75% in the F1 generation and 100% in the F2 generation through specific amplification in RT-PCR. The confirmation of CPMMV in the whole seed and seed parts harvested from F1 generation plants and the seedlings derived from F1 seed strongly support the vertical transmission of the virus. This high rate of CPMMV transmission among the germinated seedlings might be due to invasion of the virus during embryonic development as observed in other seed transmitted plant viruses (Wang and Maule, 1994; Sutrawati et al., 2021). These findings aligned with earlier studies which reported seed transmissibility of CPMMV in soybean (Thouvenel et al., 1982; Iwaki et al., 1986). In Indonesia, seed transmission rates for CPMMV ranging from 27 to 86% have been reported across different soybean cultivars and generations (Sutrawati et al., 2021). Similarly, seed transmission percentage of 3.1 to 10.75% was reported in Egypt on two soybean genotypes under field and screen house conditions (El-Hammady et al., 2004). In India, a carlavirus causing severe mottle in groundnut was found to be seed-transmissible at a rate of 4.2–7.0% in seeds from sap inoculated plants (Prasad et al., 1990). A study done on necrotic isolate of CPMMV in Brazil, performed a grow out test with 2,000 seeds in which not even a single symptomatic plant was obtained, suggesting the absence of seed transmission under those conditions (Almeida et al., 2005). The symptoms observed on seedlings in grow-out test consisted of necrotic and chlorotic spots and blotches rather than veinal necrosis. Symptom development is known to vary with temperature, humidity, and growth stage (Sandra et al., 2020b). The veinal necrosis observed in field-grown soybean may not manifest during controlled grow-out conditions due to lack of environmental stressors. Also, CPMMV can persist in seeds and infect emerging seedlings without inducing visible symptoms, reflecting latency or host tolerance during early infection (Sutrawati et al., 2021). With the reducing titer value of virus in next generation as observed in real-time PCR, it might not be able to produce similar, strong symptoms as produced in seeds. These varying rates highlight the role of host genotype, environmental conditions, and virus isolate on seed transmissibility of CPMMV.

RT-qPCR is a powerful molecular tool offering high sensitivity and specificity for detecting plant viruses in seed tissues. It enables rapid identification of viral RNA, even at low concentrations, making it ideal for screening of seeds for infection. This technique has been used in multiple studies for detection and quantification of seed-transmitted viruses, including SYMMV (Sandra et al., 2020a), *tobacco mosaic virus* (Ellis et al., 2020), *cucumber green mottle mosaic virus* (Xinying et al., 2022), and *tomato brown rugose fruit virus* (Ota et al., 2025). In the present study, RT-qPCR investigation revealed that the naturally infected soybean seeds had the greatest viral titers, showing that CPMMV was efficiently transported and accumulated within the reproductive tissues throughout seed development highlighting the importance of seed as a potential inoculum source in disease epidemiology. F1 seeds obtained from plants grown from field infected soybean seeds retained substantial viral loads, but lower than those in the field infected seeds. This might be due to a dilution effect, partial exclusion mechanisms in seed tissues or due to variations in virus transit through the phloem or funiculus (Carroll, 1981; Sastry, 2013). Analysis of cowpea seeds through RT-qPCR harvested from sap inoculated plants exhibited the low viral titers suggests that systemic transport of CPMMV is less efficient in tested cowpea genotypes. This might be due to dependence of virus replication and systemic movement on host factors, host-specific resistance responses or degree of host-virus incompatibility (Horn et al., 1993). Virus distribution among different cowpea seed components, i.e., seed coat, cotyledons, and embryo, showed variability and no definitive pattern was observed. However, whole seeds generally showed higher viral load than seed parts, which might be due to cumulative viral load across all seed tissues.

The plant and seed developmental stages are negatively impacted by the virus infection (Escalante et al., 2024). In this investigation, a large number of virus-infected plants died and did not develop pods. Even if they produced pods, the number of pods and seed size was reduced. Similar results were also observed in CPMMV infected soybean cultivars in Brazil (Da Silva et al., 2020). Assessment of seed quality parameters in the two infected genotypes, Asb-114 and AMS-2022-1, revealed significant decline in germination percentage, seedling vigor indices, fresh weight and 100-seed weight as compared to healthy controls. The reduction in germination percentage likely indicates impaired seed viability due to virus induced physiological disruptions. Furthermore, reduced seedling length and fresh weight in infected seeds emphasize the negative impact of viral infection on early seedling vigor, potentially due to constraints on cellular metabolism and resource allocation (Nallathambi et al., 2020). The observed decline in test weight might be due to interference of viral infection with nutrient mobilization or seed filling processes during seed development especially in legumes (Mandhare and Gawade, 2025).

In the present study, CPMMV infection caused distinct biochemical alterations in soybean seedlings, especially distinguished by oxidative stress and modulation of key defense related enzymes. Infected seedlings exhibited significantly elevated levels of H₂O₂, indicative of an oxidative burst commonly associated with early plant immune responses to viral invasion. A comparable oxidative burst, involving H₂O₂ accumulation, alterations in redox enzyme activities and peroxisome proliferation, has been documented in wheat (*Triticum aestivum*) infected with wheat streak mosaic virus (Mishchenko et al., 2021). Increased CAT and PAL activities in CPMMV infected seedlings suggest the activation of antioxidant and phenylpropanoid defense pathways. PAL up regulation is particularly significant as it promotes biosynthesis of lignin and related phenolic compounds that contribute to structural reinforcement and antiviral defense (Kumar et al., 2020). Although POX, SOD and total phenolic content showed slight elevations in infected tissues, their changes were not statistically significant, indicating that virus-induced enzymatic responses may be influenced by specific virus–host interactions. Similar defense related biochemical modulations have been reported in pumpkin (*Cucurbita pepo*) infected with tomato leaf curl Palampur virus, where substantial increases in total phenol content (72% in leaves, 300% in fruits) and activities of SOD, ascorbate peroxidase (APX), guaiacol peroxidase (GPX), and CAT were observed (Jaiswal et al., 2013).

Our findings provide compelling evidence that the veinal necrosis and bud blight isolate of CPMMV is vertically transmissible through seeds up to two generations in soybean. Among the diagnostic approaches evaluated, progeny testing proved to be the most reliable method for confirming CPMMV seed transmission across successive generations. The study also highlights the deleterious effects of CPMMV infection on seed quality parameters, including germination, vigor, and morphology. Given these implications, further investigation is needed to identify resistant and susceptible genotypes to this necrotic CPMMV isolate, which could be harnessed in resistance breeding programs. Additionally, expanded surveys and seed transmissibility assessments of CPMMV in other economically important leguminous crops are essential to develop phytosanitary protocols. Collectively, this study presents the first comprehensive evidence based on serological (DAC-ELISA), molecular (RT-PCR and RT-qPCR) and grow out test based analysis of seed transmission of the CPMMV-VNB isolate, emphasizing the critical role of seed borne inoculum in the epidemiology and management of viral diseases in legume agro ecosystems.

## Data Availability

The original contributions presented in the study are included in the article/Supplementary material, further inquiries can be directed to the corresponding author.
