# Supplementary material for: Seed transmission of Carlavirus vignae (Cowpea mild mottle virus): a hidden driver of veinal necrosis and bud blight disease in soybean (Glycine max) in India
Source: Front Microbiol. 2025 Sep 16;16:1654471. doi: 10.3389/fmicb.2025.1654471 (PMC12479549; doi:10.3389/fmicb.2025.1654471)
Supplement: Supplementary file 1 [file Data_Sheet_1.DOCX]

**a) Formula for the estimation of viral copy number**

Copy no. can be calculated by using the formula:

Copy No./μL of sample = Conc of Plasmid DNA(gm/ μL)× Avogadro No

Molecular weight of plasmid

Molecular wt of Plasmid =Mol. Wt. of TA cloning Vector + Mol Wt of CP gene

= (Total no of bases) × Mol Wt of one base× No of DNA strands

= (2723bp +178bp) × 330 × 2

= 1,914,660 g

Conc of Plasmid = X ng / μL

= X × 10^-9^g/ μL

Copy No. / μL of sample = X × 10^-9^ (gm / μL) × 6.023× 10^23^

1,914,660 g

**b. Seedling vigour Index calculation as per Abdul Baki and Anderson, 1973**

Seedling Vigour Index I (SVI-I) = Seed Germination %× Seedling length (cm)

Seedling Vigour Index II (SVI-II) = Seed Germination %× Seedling dry weight (g)
